# A rapid and high sensitivity RNA detection based on NASBA and G4-ThT fluorescent biosensor

**DOI:** 10.1038/s41598-022-14107-y

**Published:** 2022-06-16

**Authors:** Jia Guoshuai, Xu Xiaomeng, Guo Zengdan, Hu Xingxing, Pan Qi, Zhu Hanbing, Wang Yi

**Affiliations:** College of Life Sciences, South-Central Minzu University, Wuhan, 430074 People’s Republic of China

**Keywords:** Biological techniques, RNA

## Abstract

In recent years, various newly emerged and re-emerged RNA viruses have seriously threatened the global public health. There is a pressing need for rapid and reliable nucleic acid–based assays for detecting viral RNA. Here, we successfully developed a highly sensitive, easy-to-operate G4-ThT-NASBA system to detect viral RNA that no need for labeled primers and probes. Next, we tested the system for detecting the Classical Swine Fever Virus (CSFV), an RNA virus that causes a highly contagious disease in domestic pigs and wild boar and easily causes huge economic losses. Results showed that the system, integrated the G4-ThT fluorescent biosensor and NASBA (Nuclear acid sequence-based amplification),is capable to detect as little as 2 copies/μL of viral RNA without interfering by other swine viral RNA. Moreover, we were able to detect CSFV RNA within 2 h in serum samples taken from the field in a real-time mode. These findings indicate that the G4-ThT-NASBA system is a rapid, high sensitivity and easy-to-operate technique for RNA detection. The method also has the real-time detection capability which may be easily integrated in a highly automated system such as microfluidic chips.

## Introduction

As one of the biggest threats to global public health, viruses have received widespread attention. In which RNA viruses have brought many challenges to the existing detection and prevention system due to their high mutation rate and frequent recombination^1^. Early virus detection methods mainly used the isolation and culture of the virus from the tissues, blood, etc. of infected individuals. The identification of the virus usually took at least a week^2^. The method is not only time consuming, but also easy to contamination and misdiagnosis. The diagnosis based on Nucleic Acid Amplification Technology (NAT) can overcome these disadvantages. RT-qPCR has been widely used in RNA virus diagnosis due to its unique advantage. But it mainly disadvantage is in the need for expensive thermal cyclers and fluorophore-labeled probes^3^. The emergence of nucleic acid isothermal amplification technology and new biosensors could overcome these disadvantages.

NASBA is a sensitive transcription-based isothermal amplification system (TAS) for the specific in vitro amplification of nucleic acids^4^. This isothermal amplification reaction involves three enzymes: AMV Reverse Transcriptase (RT), RNase H and T7 RNA polymerase. This method is suitable for RNA analysis, such as mRNA, rRNA or genomic RNA. NASBA has been used in viral nucleic acid detection due to its advantages such as the combination of reverse transcription and amplification processes and the needless for temperature-changing instruments. And it has shown high sensitivity and accuracy^5,6^. Moreover, NASBA also shows some advantages in cooperation with more sensors, including Enhanced Chemiluminescence (ECL) ^7^, Enzyme-linked Oligonucleotide Capture (EOC)^8^, Molecular Beacons (MB) ^9^ and so on.

In recent years, G-quadruplex as a specific nucleic acid structure has been used for molecular diagnosis^10,11^. It has been found that the combination of G-quadruplex and Thioflavin T can not only promote the folding of the G-quadruplex structure, but also increase the fluorescence intensity of ThT by more than 1700 times^12^. Compared with other G-quadruplex sensors, this sensor significantly reduces the dependence on K+ which is the limitation of G-quadruplex as a detection sensor. Moreover, this kind of sensor has more fluorescence response in G-quadruplex RNA than DNA^13^ and is more suitable for construction of NASBA-based detection methods. In this study, we developed a fluorescence detection method combined with the NASBA and G4-ThT fluorescent sensor which can easily detect viral RNA sensitively and specifically.

Classical Swine Fever Virus (CSFV) is a single-stranded positive RNA virus that can cause highly infectious animal diseases. CSFV has long existed in developing countries and often causes huge economic losses^14^. In this study, we took CSFV as the detection object by using the system integrated the G4-ThT fluorescent biosensor and NASBA. It can be achieved sensitive and specific diagnosis of viral RNA in serum within 2 h. Meanwhile, we have also verified the possibility of this method for real-time fluorescence detection, which further increases the practicability of this system. This study provides a new solution for rapid, convenient and instant field diagnosis of viral RNA.

## Results

### Detection mechanism

**Figure 1:**
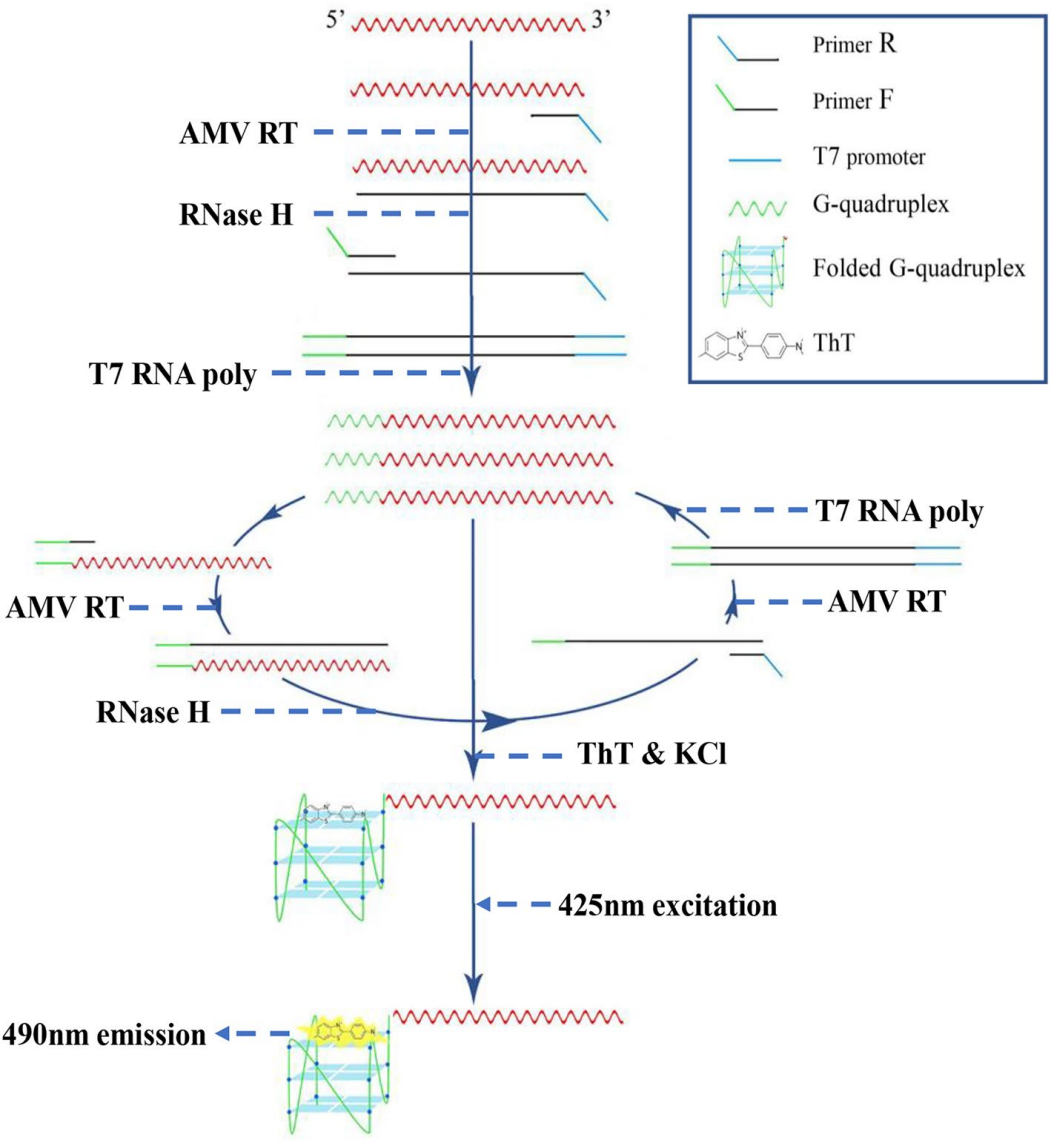
Principle of target RNA detection by G4-ThT-NASBA system. The G4-ThT-NASBA system features the addition of a G-quadruplex reverse complementary sequence on primer F, thereby generating an RNA product containing a G-quadruplex.These products bind to ThT with a fluorescence response detected at 425 nm excitation at 490 nm emission.

Figure 1 shows the principle of the G4-ThT-NASBA system. In this method, we designed a pair of primers. Primer F contains CSFV-E2 gene binding region and T7 promoter, and primer R contains CSFV-E2 gene binding region and G4 reverse complementary sequence. The specially designed primers and the template are subjected to NASBA reaction, and a large number of G-quadruplex fragments can be obtained. The addition of ThT promotes the folding of the G-quadruplex. After being irradiated with 425 nm excitation light, a significant fluorescence increase can be detected at 490 nm.

**Figure 2:**
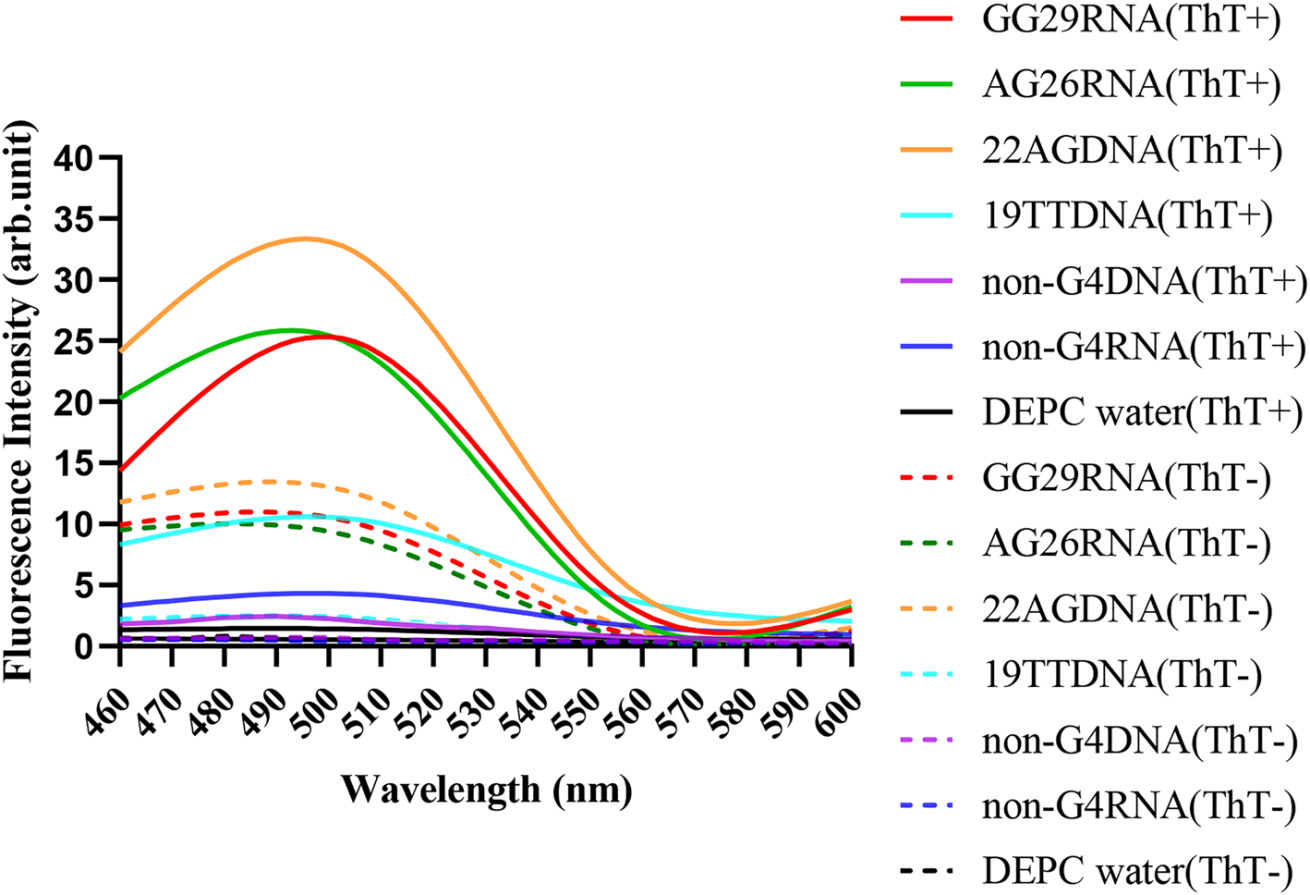
Construction of G4-ThT fluorescence biosensor. ThT fluorescence spectra of G-quadruplex and non-G-quadruplex in NASBA system. Conditions: GG29 RNA, AG26 RNA, 22AG DNA, 19TT DNA, non-G4 DNA, non-G4 RNA final concentration, 3 μM; dyeing time, 10 min; ThT−: no ThT added (dotted line); ThT+: final concentration of ThT, 3 μM (solid line).

In order to verify the feasibility of G-quadruplex as a biosensor, we added different G4 RNA or DNA and non-G4 RNA or DNA to the NASBA reaction system to detect its fluorescence spectra. As shown in Fig. 2, GG29RNA, AG26RNA and 22AGDNA can have significant fluorescence enhancement at 490 nm emission light under 425 nm excitation light, and the addition of ThT enhances this fluorescence behavior. Other non-G4 sequence DNA or RNA showed no obvious fluorescence enhancement. Therefore, the G4-ThT sensor can be used to detect NASBA amplification products with G4.

### Condition optimization of the method

To achieve the best performance of the detection method, we have optimized some key factors in the detection process, including the primers, the concentration of ThT and K^+^ in the system. Three NASBA R primers and five F NASBA primers with different G-quadruplex reverse complementary sequences were optimized. In Fig. S1A, it can be seen that among the three reverse primers, T7R2-1 and T7R2-3 produce high background fluorescence values while T7R2-2 produces lower background fluorescence. So T7R2-2 is selected as the reverse primer. The F primers of the five different G-quadruplexes did not produce background fluorescence. The amplification products of different G4-labeled forward primers were analyzed, and the appropriate NASBA forward primers were determined according to the relative fluorescence values. As shown in Fig. S1B, comparing with the different primers, the relative fluorescence difference of the amplified product of F2-2 GG29 primer was higher than the other four groups.

The concentration of ThT significantly affect the detection sensitivity of the system. As shown in Fig. S2, the increase of ThT concentration within a certain range will increase the relative fluorescence value of positive amplification products. But it will also increase the background fluorescence in the negative product.

The presence of K^+^ generally promotes the folding of the G-quadruplex. The results are shown in the Fig. S3, the addition of additional K^+^ will not have a significant effect on the background fluorescence in the negative product. In the positive product, we found that too much K^+^ not only does not increase the fluorescence value, but also produces some inhibitory effects. After a series of optimization experiments, we finally selected F2-2 GG29 as the forward primer for the NASBA reaction. The ThT concentration was selected as 4 μM and no additional K^+^ for the following experiments.

### Sensitivity and specificity for CSFV detection

**Figure 3:**
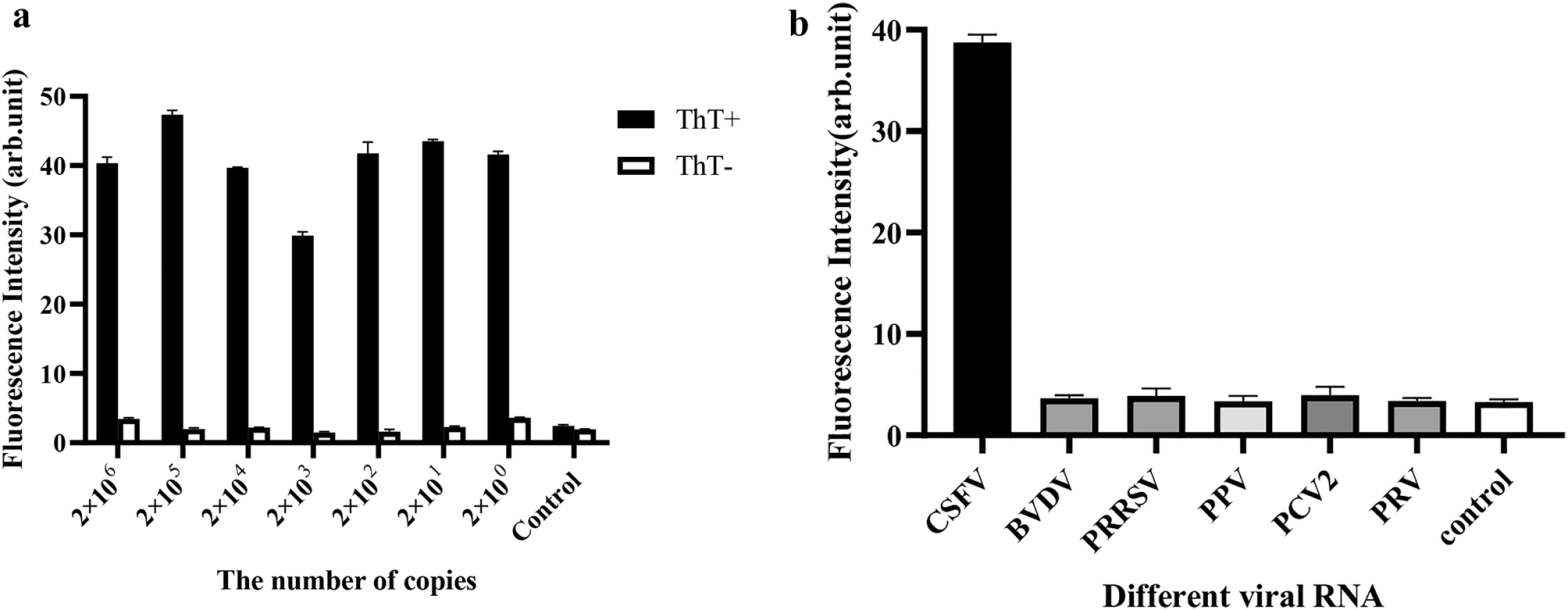
Sensitivity (**a**) and specificity (**b**) for CSFV detection by G4-ThT-NASBA system. (**a**) Detection sensitivity. Amplification products with different copies CSFV-E2 RNA use ThT to detect. The fluorescence values were measured at 425 nm excitation and 490 nm emission and normalized to the value of negative control. Negative control: the group with no template added to the detection system. The data represent the mean ± S.D. of three independent experiments. (**b**) Detection specificity. Total RNA extracted from different viruses infected cells were detected for CSFV RNA. The fluorescence values were measured at 425 nm excitation and 490 nm emission and normalized to the value of negative control (Ct). Ct: negative control with no template added to the detection system. The data represent the mean ± S.D. of three independent experiments.

To test the sensitivity of the system, we explored the fluorescence response with different concentrations of CSFV-E2 RNA. In Fig. 3a, compared with the negative control, the relative fluorescence intensity of the different copy number RNA samples and the negative control reached at least to more than 15 times enhancement. However, when ThT was not added to the system for detection, we did not find the fluorescence response in the system. We judged that these fluorescent responses came from the reaction between ThT and the product. Compared with the previously reported CSFV nucleic acid fluorescence detection method^15^, we found that the G4-ThT-NASBA system achieved a lower detection limit.

To test the specificity of the system, we selected BVDV, PRRSV, PPV, PCV2 and PRV and other porcine viruses for analysis. In Fig. 3b, all other swine viruses showed the same fluorescence intensity level as the negative control group. They were all significantly lower than (20 times) the relative fluorescence of the CSFV group (positive control). The above results indicate that the G-4-ThT-NASBA system has a good specificity for CSFV detection.

### G4-ThT-NASBA real-time test

**Figure 4:**
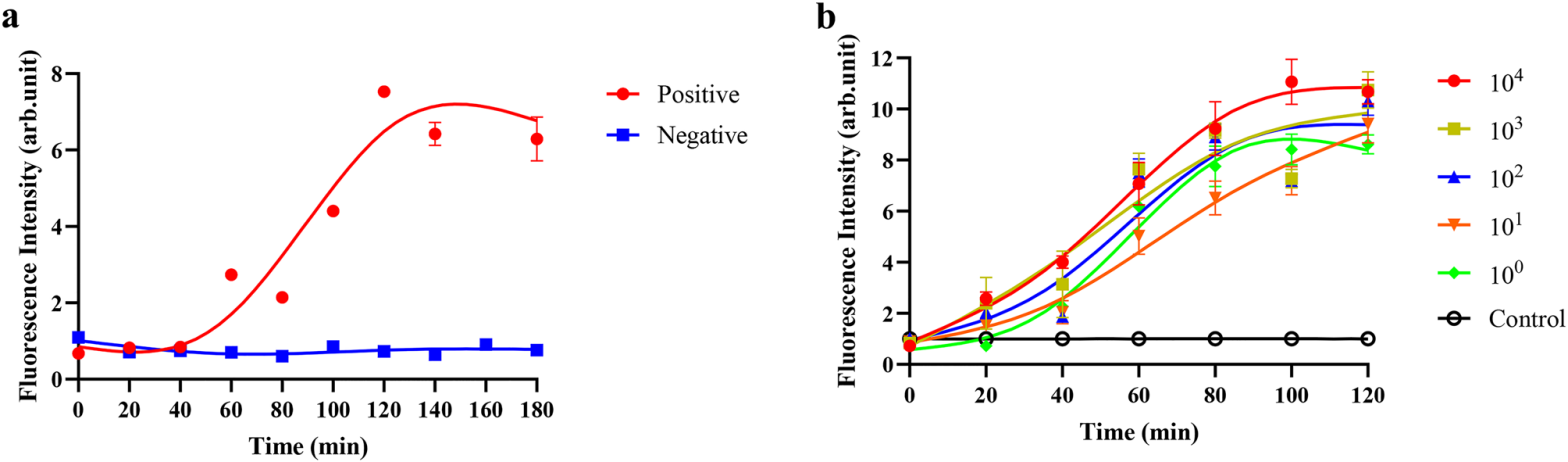
G4-ThT-NASBA real-time system detect CSFV-E2 RNA. (**a**) Fluorescence curve of CSFV-E2 RNA detected by real-time G4-ThT-NASBA system. Draw a curve according to the fluorescence value of the product at different reaction times. NASBA primer R2 and primer F GG29 with CSFV-E2 RNA are shown in red (positive control); NASBA primer R2 and primer F GG29 with no template are shown in blue (negative control). (**b**) Detection sensitivity of G4-ThT-NASBA real-time system. Different copies of CSFV-E2 RNA are represented by different colored fluorescence curves.

We conducted research on real-time fluorescence detection using G4-ThT-NASBA system. The results shown in Fig. 4a, the results suggest that in the real-time fluorescence test, the relative fluorescence value of the positive samples decreased compared with the previous end-point method. For the sensitivity of the system, the ThT concentration used in the real-time fluorescence detection was re-optimized. The curves by the real-time fluorescent G4-ThT-NASBA system to detect CSFV E2 RNA at different ThT concentrations are shown in Fig. S4. After comprehensive comparison, the optimal ThT concentration was determined to be 6 μM. As shown in Fig. 4b, the system maintained the same sensitivity as the previous end-point method, and the samples all produced fluorescence enhancement within 40 min. In these results, we found that the addition of ThT in advance reduced the final detected fluorescence level, which we speculated was caused by the prolonged reaction time of the G4-ThT sensor, but these reductions did not affect whether the system detected whether the template contained target RNA.

### CSFV detection of cells and serum samples

**Figure 5:**
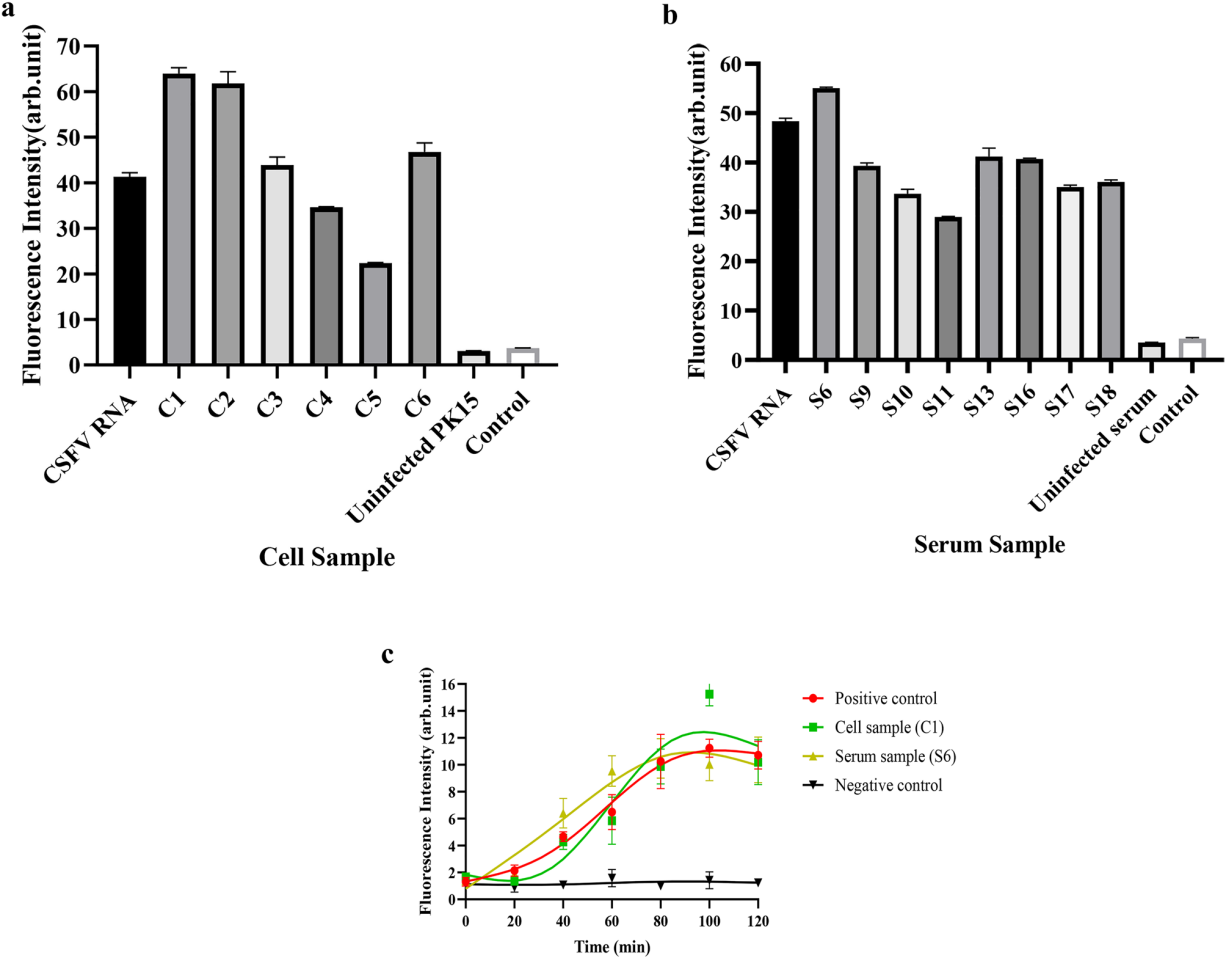
Detection of CSFV-infected cells and clinical pig serum by G4-ThT-NASBA system. (**a**) Detection of CSFV in cell samples (C1–C6 & uninfected PK15) CSF viral RNAs. (**b**) Detection of CSFV in serum samples (S6, S9, S10, S11, S13, S16, S17, S18 and uninfected serum) CSF viral RNAs. The fluorescence values were measured at 425 nm excitation and 490 nm emission and normalized to the value of negative control (Ct). Ct: negative control with no template added to the detection system. (**c**) The fluorescence change curve of CSFV-E2 RNA detected by real-time fluorescent G4-ThT-NASBA system is shown in red (Positive control), and the fluorescence change curve of detection without template is shown in black (Negative control). The fluorescence change curve of CSF virus RNA in cell specimens It is shown in green (Cell sample C1), and the detection fluorescence curve of CSF virus RNA in serum samples is shown in yellow (Serum sample S6).

We used this system to detect viral RNA in CSFV-infected PK15 cells and serum samples. The results are shown in Fig. 5a, the relative fluorescence value of the positive control group was more than 20 times that of the negative control group. Among the 6 CSFV-infected cell samples, the relative fluorescence value of the C1 sample was the highest, reaching 20 times that of the negative control group. The relative fluorescence value of the C5 sample reached at least 8 times that of the negative control group. However, the uninfected PK15 group maintained the same fluorescence level as the negative control group. The results of the clinical blood sample are shown in Fig. 5b. After the total RNA of 8 swine serum samples was analyzed, the relative fluorescence value of the S6 sample was the highest and reached 20 times that of the negative control group. The lowest fluorescence value of the S11 sample was 15 times that of the negative control group. The uninfected pig serum samples had the same fluorescence level as the negative control group.

Figure 5c shows the fluorescence curves obtained by detecting the CSFV-infected PK15 cell samples (C1) and blood samples (S6) by real-time mode. The amplification curve for the positive cell samples produces fluorescence enhancement at about 40 min, which is slightly slower than that of the positive control. While the curve for the positive serum samples is at 20 min. Compared with the negative control samples, the real-time G4-ThT-NASBA system can detect the presence of CSFV target RNA in cell samples and blood samples.

## Discussion

New and re-emerging viruses are responsible for many recent epidemics. Among these viruses, RNA viruses have brought many challenges to virus detection, prevention and control due to their high mutation rate and high adaptability^16^. In this study, we developed a novel label-free G4-ThT-NASBA system that could specifically detect CSFV viral RNA. Results showed that this system could detect viral RNA at a minimum of 2 copies/μL and without interfering by other porcine viral RNAs. We also tested the possibility of this system for real-time fluorescence detection. Results showed a sensitivity and specificity equivalent to that of the endpoint method which further expands the application scenarios of this system.

At present, most specific nucleic acid detection methods rely on the hybridization between the probe DNA and the target sequence, and then signal amplification and detection by electrochemistry^17^, colorimetry^18^ or fluorescence^19^. However, these methods need label various tags to nucleic acid sequences to make photoelectric biosensors, which are not only expensive but also difficult to store and thereby limit their application in field detection. As a specific nucleic acid structure, G-quadruplex has been confirmed to realize the nucleic acid detection without chemical label. The split G-quadruplex probe was used to carry out the signal output of the oxidation reaction through the peroxidase properties of the complete G-quadruplex^20^. Regardless of whether it is based on G-quadruplex peroxidase enzyme activity or fluorescence characteristics, most of the current methods rely on the form of split probes or molecular beacons^15,21,22^. In this study, the detection method of G-quadruplex generated during isothermal amplification has not been reported before.

The latest research showed that the fluorescent complex ThT could promote the folding of different G-quadruplex DNA or RNA^23^. We use the feature to construct and optimize a new biosensor G4-ThT, and combine the sensor with the isothermal amplification method NASBA to develop a new viral RNA detection system. In the past, the detection of CSFV, an RNA virus, mainly relied on virus isolation and serological diagnosis. At present, molecular diagnosis such as RT-qPCR have also been used in CSFV detection. However expensive thermal cyclers and labeled probes are often required, which increases the cost of detection and is not convenient for detection in remote areas^24^. In addition, Abdolahzadeh et al. used the combination of nested NASBA and Mango aptamers to construct an RNA detection method^25^. But this method needs two NASBA reactions, which is not very convenient and may be easy contaminated. The G4-ThT-NASBA detection system in this study could overcome these disadvantages very well.

In conclusion, the G4-ThT-NASBA system provides a more convenient, economical and wider application scenario for the detection of viral RNA. This method is more suitable for transplantation on microfluidic chips and other automated systems due to its simple amplification steps and real-time detection mode.

## Materials and methods

### Primer and probe design

According to the comparative analysis of the CSFV-E2 sequence on NCBI, combined with the reverse complementary sequence of the G-quadruplex^23,26^ mentioned in the previous study, the PCR and NASBA amplification primers designed according to the primer design principle are shown in Table 1.

**Table 1:** List of primers and probes for CSFV detection by G4-ThT-NASBA system.

Primers	Sequence (5′–3′)	Description
CSFV E2 primer F	AATTCTAATACGACTCACTATAGGGAGAGGA*CTACCACCTGGAAAGAATAC*	Product size: 1233 nt Underline sequences are T7 promoter; italic sequences can complementary to the target
CSFV E2 primer R	*TAACCATAAGCAATGCCACT*	
NASBA primer R1	AATTCTAATACGACTCACTATAGGGAGAGGA*GCGGCGAGTTGTTCTGTTAG*	Underline sequences are T7 promoter; italic sequences can complementary to the target RNA
NASBA primer R2	AATTCTAATACGACTCACTATAGGGAGAGAA*CGGCGAGTTGTTCTGTTA*	
NASBA primer R3	AATTCTAATACGACTCACTATAGGGAGAGA*GCGAGTTGTTCTGTTAGA*	
NASBA primer F	*TCCTGTGGCTAATAGTGACC*	
NASBA primer F GG29	CCCCTACCTCCCGCCCCTACCCGTCCCCC**TCCTGTGGCTAATAGTGACC**	Underline sequences are G-quadruplex reverse complementary sequence; bold sequence can complementary to the target RNA
NASBA primer F 22AG	CCCTAACCCTAACCCTAACCCT**TCCTGTGGCTAATAGTGACC**	
NASBA primer F BCL2	CCCCAGCTCCCACCCCACGGCCCCCT**TCCTGTGGCTAATAGTGACC**	
NASBA primer F TRF2	GCCCTCCCCGCCCTCCCG**TCCTGTGGCTAATAGTGACC**	
NASBA primer F VEGF	TCCTCCTCCCCCTCCTCC**TCCTGTGGCTAATAGTGACC**	
GG29 RNA	GGGGGACGGGTAGGGGCGGGAGGTAGGGG	Italic sequences are G-quadruplex RNA, bold sequences are G-quadruplex DNA
AG26 RNA	AGGGTTAGGGTTAGGGTTAGGG	
22AG DNA	**AGGGTTAGGGTTAGGGTTAGGG**	
19TT DNA	**TGGGTTGGGTTGGGTGGGT**	

### CSFV-E2 target RNA generation

The CSFV E2 DNA is derived from the full-length infectious clone of CSFV pT7SM plasmid deposited in our laboratory. Using the CSFV E2 F and R primers in Table 1, the pT7SM plasmid was used to amplify the E2 segment with the T7 promoter according to the procedure shown in the instruction manual of Gold MIX Ver.2 (Tsingke, TSE102). The above PCR product fragments were purified using Takara MiniBEST DNA Fragment Purification Kit (Takara, China). Use 1 μg of purified PCR fragment, T7 RNA polymerase (Thermo Scientific), in ATP/GTP/CTP/UTP Mix (2 mM final concentration), 5 × Transcription buffer for in vitro transcription at 37 °C for 2 h; after the reaction, add 2 U DNaseI, RNase-free (Thermo Scientific), 37 °C for 15 min to remove template DNA. The concentration of RNA synthesized in vitro was measured with a NanoDrop 2000c spectrophotometer (Thermo Science, USA). The measured RNA mass concentration was converted into copy number concentration by the RNA copy number calculation formula, and dilute to target RNA with different copy number concentration for using.

### Viruses, cells and serum

The cells infected with 1 CSFV Shimen strains, 1 BVDV strain (NADL), 1 PRRSV strain (Hubei), 1 PRV strain (HB), 1 PPV strain (HN99), 1 PCVII strain (HBZX) were provided by Hubei Academy of Agricultural Sciences. Clinical swine serum samples infected with CSFV are preserved in our laboratory.

### Extraction of viral RNA

Trizol (Invitrogen, CA, USA) method was used to extract total RNA from cell and serum samples, according to the manufacturer’s protocol. The purity of the extracted total RNA was determined by measuring the absorbance ratio at 280 nm and 260 nm, and the RNA concentration was based on the absorbance at 260 nm using a Nanodrop 2000c spectrophotometer (Thermo scientific, USA). RNA samples with a ratio of 260 nm/280 nm of 1.9–2.1 were used for NASBA templates. Ten-fold serial dilutions of RNA from virus isolates were prepared.

### Detection of G-quadruplex and ThT fluorescence spectroscopy

Different G-quadruplex RNA and DNA probe sequences were sent for synthesis, and the sequences are shown in Table 1. The different G4 and non-G-quadruplex probes were heated to 80 °C for denaturation for 5 min, slowly cooled to room temperature. The final concentration of detection probe was 3 μM, the final concentration of KCl was 50 mM, and the final concentration of ThT was 3 μM for ThT fluorescence detection. Fluorescence response in the absence of ThT for each G4 RNA, DNA and non-G4 DNA, RNA. The reaction mixture was placed in a 96-well black microplate, and the fluorescence value was detected every 10 nM under the excitation light of 425 nm and the emission light of 460–600 nm, and the fluorescence spectrum was drawn using Graphpad prism 8 according to the relative fluorescence value. The data represent the mean ± S.D. of three independent experiments.

### NASBA procedure

The NASBA procedure used in this study was previously described by kievits ^27^. The final volume of the reaction mixture was 25 μL. First, assemble 17.4 μL volume of pre reaction mixture comprising: 50 mM Tris/HCl(pH 8.5), 10 mM MgCl_2_, 90 mM KCl, 10 mM DTT, each dNTP 1 mM, each NTP 2 mM, 10% (v/v) DMSO, 0.4 μM NASBA primer F and 0.4 μM NASBA primer R. Add 2 μL target RNA, the mixture was incubated at 65 °C for 5 min to unfold the secondary structure of the target RNA, and the reaction mixture was transferred to 41 °C for annealing for 5 min. Finally, add 5.6 μL enzyme mixture(4 mM DTT, 2 μg BSA, 30 U T7RNApoly(Thermo Scientific, USA), 6 U AMV-Rt(Promega, China), 0.1 U RNase H(Thermo Scientific, USA)) to the tube and obtain total volume to 25 μL. The mixture was incubated at 41 °C for 2 h for isothermal amplification of RNA targets. The amplified product can be directly fluorescence detected by ThT in the next step.

### NASBA product end-point fluorescence detection

For CSFV RNA fluorescence detection, the above NASBA reaction product was denatured at 80 °C for 3 min, slowly cooled to room temperature, and the final volume was adjusted to 71 μL with DEPC water. 4 μL of 100 μM ThT was then added to the system and incubated at room temperature for 10 min. The mixture was placed in a 96-well black microplate, and the fluorescence value at 490 nm absorption light was detected using the SpectraMax i3x Platform (Molecular Devices, LLC.) at 425 nm excitation light.

### Real-time G4-ThT-NASBA system detects CSFV RNA

In addition to the required 50 mM Tris/HCl (pH 8.5), 10 mM MgCl_2_, 90 mM KCl, 10 mM DTT, 1 mM each dNTP, 2 mM each NTP, 10% (v/v) DMSO, 0.4 μM NASBA Primer F and 0.4 μM NASBA primer R, add ThT at a final concentration of 6 μM to the reaction premix. After adding target RNA, denaturation at 65 °C and annealing at 41 °C, the enzyme premix was added and incubate at 37 °C for 0, 20, 40, 60, 80, 100, 120 min. The products of different reaction times were added to 384-well microplates, and the fluorescence value under 490 nm absorption light was directly detected under 425 nm excitation light.

### Statistical analysis

The fluorescence value results of the added reactants (positive, sample and negative) were excluded from the effect of the fluorescence value of the microplate without the added reactant. Each experiment was carried out 3 times, and the data were expressed as mean ± standard deviation. Column analysis of different experimental groups, standard error analysis of the mean value of three replicate experimental data, and Fit spline/LOWESS analysis of real-time fluorescence numerical curve were performed using GraphPad Prism version 8.0.2 for Windows, GraphPad Software, San Diego, California USA, www.graphpad.com.

## Supplementary Information

Supplementary Figure S1.

Supplementary Figure S2.

Supplementary Figure S3.

Supplementary Figure S4.

## Data Availability

All RNA-seq data from the primer design of this study can be downloaded through NCBI (Genome) (https://www.ncbi.nlm.nih.gov/genome/) with Sequence IDs AY775178.2 and AY805221.1. The data that support the findings of this study are available from the supplementary material of this article.
