# Supplementary material for: A rapid and high sensitivity RNA detection based on NASBA and G4-ThT fluorescent biosensor
Source: Sci Rep. 2022 Jun 16;12:10076. doi: 10.1038/s41598-022-14107-y (PMC9203706; doi:10.1038/s41598-022-14107-y)
Supplement: Supplementary file 1 — Supplementary Figure S1. [file 41598_2022_14107_MOESM1_ESM.pdf]

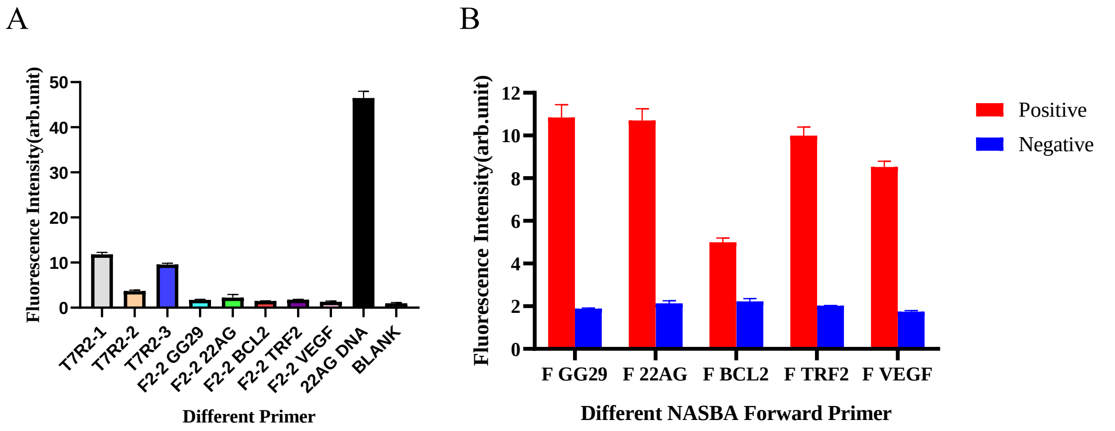

Supplemental_Fig_S1. Optimization of primers in G4-ThT-NASBA system

A: ThT fluorescence value of different NASBA primers. T7R1, T7R2, T7R3: NASBA primer R; F GG29, F22AG, F BCL2, F TRF2, F VEGF: NASBA primer F; 22AG DNA: positive control; BLANK: negative control. Conditions: primers final concentration, 3μM; dyeing time, 10 min; concentration of ThT, 3 μM. B: The fluorescence intensity of different NASBA F primers(GG29, 22AG, BCL2, TRE2, VEGF) was tested in CSFV-E2 RNA detection by G4-ThT-NASBA. The fluorescence values were measured at 425 nm excitation and 490 nm emission and normalized to the value of negative control (Ct). The data represent the mean ± S.D. of three independent experiments.
