# Supplementary material for: A rapid and high sensitivity RNA detection based on NASBA and G4-ThT fluorescent biosensor
Source: Sci Rep. 2022 Jun 16;12:10076. doi: 10.1038/s41598-022-14107-y (PMC9203706; doi:10.1038/s41598-022-14107-y)
Supplement: Supplementary file 3 — Supplementary Figure S3. [file 41598_2022_14107_MOESM3_ESM.pdf]

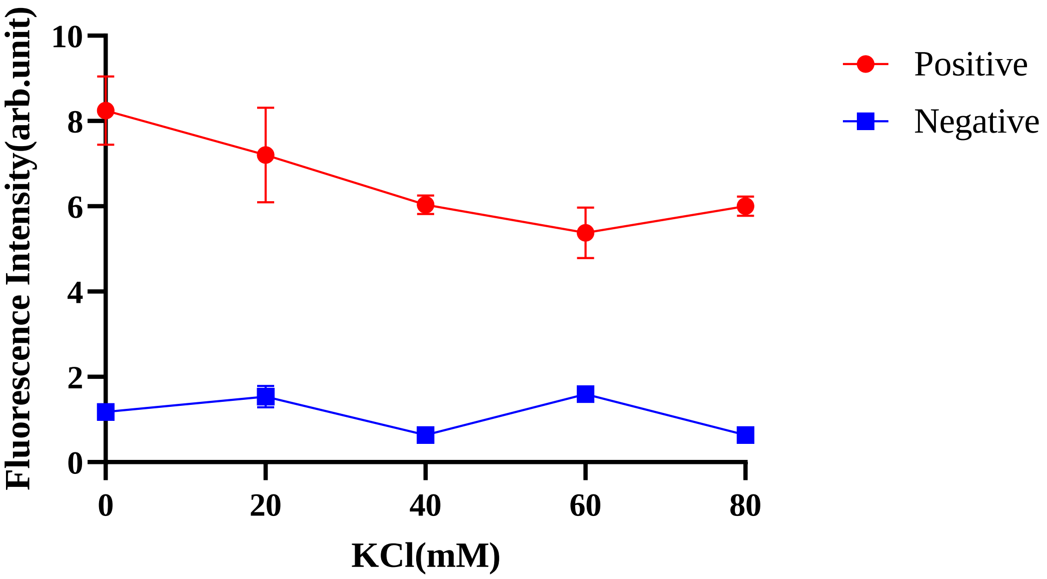

Supplemental_Fig_S3. Different concentrations (0-80 μM) of KCl were additionally added to CSFV-E2 RNA by G4-ThT-NASBA. The fluorescence values were measured at 425 nm excitation and 490 nm emission and normalized to the value of negative control (Ct). Ct: negative control with no template added to the detection system. The data represent the mean ± S.D. of three independent experiments.
