# Supplementary material for: A rapid and high sensitivity RNA detection based on NASBA and G4-ThT fluorescent biosensor
Source: Sci Rep. 2022 Jun 16;12:10076. doi: 10.1038/s41598-022-14107-y (PMC9203706; doi:10.1038/s41598-022-14107-y)
Supplement: Supplementary file 4 — Supplementary Figure S4. [file 41598_2022_14107_MOESM4_ESM.pdf]

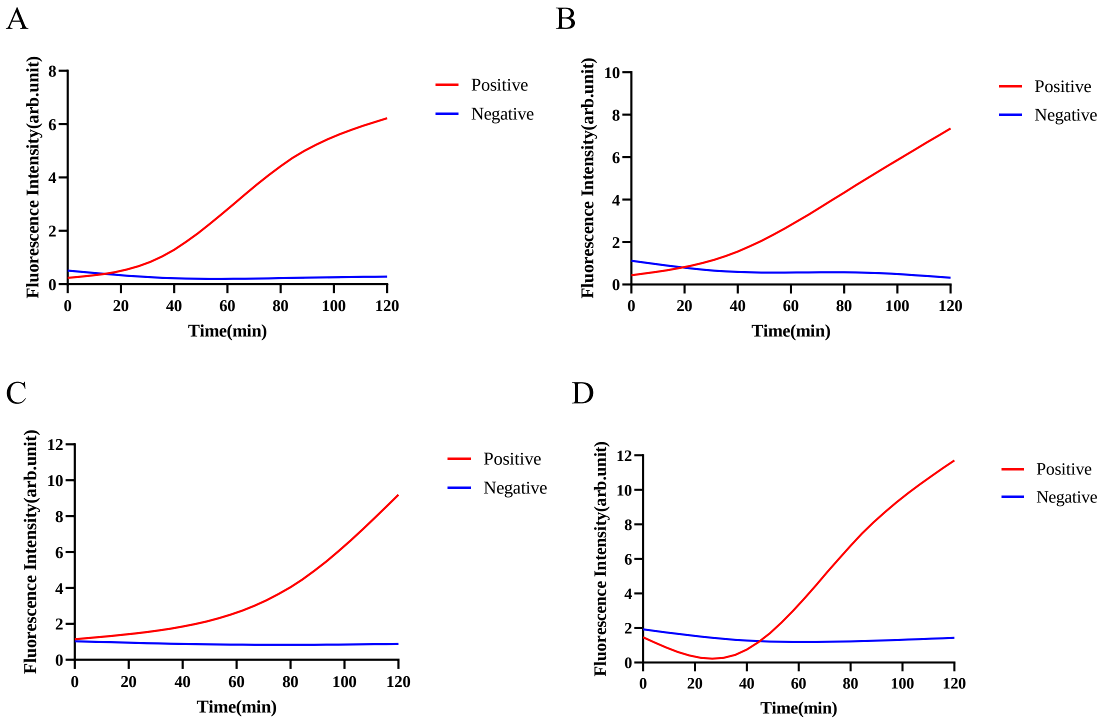

Supplemental_Fig_S4. Real-time G4-ThT-NASBA system detects CSFV fluorescence curve under different ThT concentrations. A: The final concentration of ThT is 2 μM; B: the final concentration of ThT is 4 μM; C: the final concentration of ThT is 6 μM; D: the final concentration of ThT is 8 μM.
